# Detection of motor execution using a hybrid fNIRS-biosignal BCI: a feasibility study

**DOI:** 10.1186/1743-0003-10-4

**Published:** 2013-01-21

**Authors:** Raphael Zimmermann, Laura Marchal-Crespo, Janis Edelmann, Olivier Lambercy, Marie-Christine Fluet, Robert Riener, Martin Wolf, Roger Gassert

**Affiliations:** 1, Rehabilitation Engineering Lab, ETH Zurich, Zurich, Switzerland; 2 Biomedical Optics Research Lab, University Hospital Zurich, University of Zurich, Zurich, Switzerland; 3, Sensory-Motor Systems Lab, ETH Zurich, Zurich, Switzerland; 4 Spinal Cord Injury Center, University Hospital Balgrist, University of Zurich, Zurich, Switzerland

**Keywords:** BCI, Single-trial, Hidden Markov model (HMM), Functional NIRS, Biosignals, Autonomic nervous system (ANS), Isometric pinching

## Abstract

**Background:**

Brain-computer interfaces (BCIs) were recently recognized as a method to promote neuroplastic effects in motor rehabilitation. The core of a BCI is a decoding stage by which signals from the brain are classified into different brain-states. The goal of this paper was to test the feasibility of a single trial classifier to detect motor execution based on signals from cortical motor regions, measured by functional near-infrared spectroscopy (fNIRS), and the response of the autonomic nervous system. An approach that allowed for individually tuned classifier topologies was opted for. This promises to be a first step towards a novel form of active movement therapy that could be operated and controlled by paretic patients.

**Methods:**

Seven healthy subjects performed repetitions of an isometric finger pinching task, while changes in oxy- and deoxyhemoglobin concentrations were measured in the contralateral primary motor cortex and ventral premotor cortex using fNIRS. Simultaneously, heart rate, breathing rate, blood pressure and skin conductance response were measured. Hidden Markov models (HMM) were used to classify between active isometric pinching phases and rest. The classification performance (accuracy, sensitivity and specificity) was assessed for two types of input data: (i) fNIRS-signals only and (ii) fNIRS- and biosignals combined.

**Results:**

fNIRS data were classified with an average accuracy of 79.4%, which increased significantly to 88.5% when biosignals were also included (p=0.02). Comparable increases were observed for the sensitivity (from 78.3% to 87.2%, p=0.008) and specificity (from 80.5% to 89.9%, p=0.062).

**Conclusions:**

This study showed, for the first time, promising classification results with hemodynamic fNIRS data obtained from motor regions and simultaneously acquired biosignals. Combining fNIRS data with biosignals has a beneficial effect, opening new avenues for the development of brain-body-computer interfaces for rehabilitation applications. Further research is required to identify the contribution of each modality to the decoding capability of the subject’s hemodynamic and physiological state.

## Background

In approximately one third of stroke survivors, residual hand motor function is severely impaired, even after one year of rehabilitation [1]. Currently, these patients can only be treated using passive approaches, which have been shown to be of limited efficacy [2]. There is evidence that neuroplasticity can be promoted using brain-computer interfaces (BCIs) [3]. Physical training, in which the patients are actively involved by means of a BCI, therefore has the potential to offer a novel form of active movement therapy to the severely impaired [4]. Our long-term goal is thus the development of a therapeutic intervention to restore hand function based on the automatic detection of movement intention directly from the brain, instead of relying on remaining motor function. To this end, cortical activity will be monitored and processed in real time by means of a BCI, such that an assistive robot in a virtual reality exercise environment can be controlled accordingly.

Several techniques allow for real-time monitoring of brain activity for BCI purposes. Invasive approaches have been successfully employed in non-human and human primates. Microelectrode-arrays were implanted in the cortex or electrocorticography (ECoG) was used to record brain activity, resulting in BCIs to control cursors or, more recently, prostheses [5-8]. Despite the high performance of such invasive BCIs, non-invasive methods to monitor brain activity are preferred for a rehabilitation setting. Conventional non-invasive brain recording techniques are mainly electroencephalography (EEG), magnetoencephalography (MEG), functional magnetic resonance imaging (fMRI) and functional near-infrared spectroscopy (fNIRS).

EEG is the most widely used technique employed in BCIs [9] (for a review, see [10]). However, while EEG provides good temporal and spatial resolution, the training phase required for the user to produce classifiable brain signals can be time consuming and frustrating [11]. MEG has recently been employed in a BCI to control a hand orthosis in stroke patients [1]. However, MEG is not well suited for a regular application due to its immobility, sensitivity to electromagnetic disturbances and high costs. fMRI has also been used to interface with the human brain [9,12]. Although it features high spatial resolution and whole brain coverage, the low temporal resolution, electro-magnetic compatibility constraints, high sensitivity to motion artifacts and high costs make it inappropriate in a standard therapeutic environment.

fNIRS is an optical approach that locally probes cortical activity based on the neurovascular coupling [13] and has been used in neuroimaging studies since the 1990s (for a review, see [14]). It is easy to use, safe, affordable, relatively tolerant to movement, can be miniaturized and operated wirelessly [15]. Compared to EEG, fNIRS-based BCIs allow for the classification of more naturally elicited cortical activity and require no training by the operator [11,16]. Reviews on hemodynamic BCIs can be found in [9,17].

BCIs that make use of fNIRS as a method to record brain data have been investigated for different purposes and types of classifiers. Off-line binary classification of right and left hand imagery with support vector machines (SVM) and hidden Markov models (HMM) were implemented and compared in [18]. In [19], a simple binary classification scheme was implemented on-line. The classifier was based on a direct comparison between the cortical hemodynamics during left and right motor imagery and had an information transfer rate of 1 bit/min. In these preliminary studies, the cortical response was elicited by motor tasks. Other than motor execution or imagery, mental arithmetic [20,21], music imagery [20,22], subjective preference [23] or emotional induction tasks [24] were successfully classified based on fNIRS measurements for BCI purposes. The classifiers that were employed include HMM [18,20,22], SVM [18,24], linear discriminant analysis (LDA) [23-25] and artificial neural networks (ANN) [26,27].

However, fNIRS is not only sensitive to the hemodynamic response of brain activity. The measured signals also contain physiological components [28] which might influence the classification performance of a BCI. It is well known that pulsation is visible in the fNIRS data [29]. Furthermore, respiration and slow oscillations with frequencies around 0.1 Hz (Mayer waves) are usually present in the data [30-33]. We recently reported results of a statistical off-line analysis from a pilot study carried out with seven healthy subjects, in which we showed that significant changes in oxy- and deoxyhemoglobin in the left primary motor cortex (M1) were indeed accompanied by significantly increased blood pressure, respiration rate and skin conductance during an isometric finger pinching task as compared to rest [34]. A beneficial effect of the inclusion of biosignals on the decoding performance yielding a hybrid BCI was found in EEG-based BCIs [35]. Interestingly, there are only a few studies that explicitly take these parameters into account in fNIRS-based BCIs. Biosignals were explicitly used for classification in [22], where an increase in the performance of a classification between music imagery and rest was found when including the respiration effort, heart rate (HR), skin temperature and electro-dermal activity for classification. We are not aware of any other fNIRS-based BCI study which made explicitly use of biosignals.

In this paper, offline single-trial classification of motor execution and rest in healthy subjects is presented. More specifically, we addressed the questions (i) how motor execution (i.e. isometric finger pinching) and rest periods can be decoded on a single-trial basis in healthy subjects based on fNIRS data obtained from contralateral M1 and/or ventral premotor cortex (PMv) and (ii) how the inclusion of the response of the autonomic nervous system (ANS), as acquired in the biosignals, affects the classification performance. In contrast to aiming at a BCI that generalizes to a wide range of users, an approach with subject-specifically tuned classifier topologies was opted for in order to allow for the individual optimization of the performance. Emphasis was furthermore placed on a detailed description of the methodology to analyze and process the recorded signals. To the best of our knowledge, we are the first to report the design and performance of a BCI which is intended to be used for motor rehabilitation and which is based on fNIRS data from cortical motor areas and simultaneously acquired biosignals.

## Methods

### Subjects

Seven healthy volunteers (mean age ± SD: 26.0 ± 2.2 y, 1 female) were recruited among the members of the laboratories involved in this project. Inclusion criteria were no known history of current or past neurologic, psychiatric and mental disorders as well as drug- and alcohol-related conditions. The study was approved by the institutional ethical committee of the ETH Zurich (Application Number: EK 2010-N-49) and participants gave written informed consent.

### Protocol

**Figure 1:**
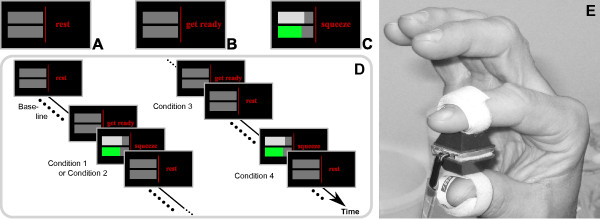
**Illustration of the task and the experimental protocol.A**: visual stimulus during the rest periods. **B**: visual cue. **C**: visual stimulus during the isometric pinching periods. Upper bar (white): desired force. Lower bar (green): achieved force. **D**: Schematic representation of the experimental protocol. Each recording session started (ended) with 180 (120) seconds of baseline, followed by random presentation of the four different conditions. Conditions were separated in time by rest periods of randomized durations (from 15 to 24 seconds). **E**: force sensor attached between the tips of the right index finger and thumb. **A-D**: Ⓒ2011 IEEE. Reprinted, with permission, from [34].

The measurements were conducted in a quiet, dimmed room. Subjects were asked to lie supine on a stretcher. The task consisted in isometrically pinching a force sensor (CentoNewton 100N, LPM-EPFL) attached between the tips of the index finger and thumb with Velcro^®^ straps (Figure 1E). For this feasibility study, we focused on overt motor tasks with the right hand, irrespective of the subject’s handedness. Isometric pinching was chosen as task in order to minimize movement artifacts and to restrict subjects to a well-defined and consistent motor execution. Subjects were asked to track a given reference force which was generated from a truncated Fourier series with frequencies 0.5, 1.0 and 1.1 Hz to reduce learning effects and ranged approximately from 1 to 4 N. Video goggles (Zetronix z920HR-VGA) were used to provide visual feedback of both the reference as well as the applied force (Figure 1C).

Three visual stimuli were presented [34]. During the rest period, the word *rest* was displayed (Figure 1A) and subjects were requested to relax their mind and body. A visual cue was provided as shown in (Figure 1B). The expression *get ready* appeared on the screen and subjects were asked to prepare for motor execution. During the pinching period, two horizontal bars were visible that showed the measured applied force (Figure 1C, upper bar) as well as the reference force (Figure 1C, lower bar). The word *squeeze* was further displayed and subjects were requested to perform the described motor task.

Four different *conditions* as illustrated in Figure 1D were derived from the three visual stimuli: 

• Conditions 1 and 2 were defined as cued motor executions (rest–cue–isometric pinching–rest), with cue durations of 10 and 5 seconds, respectively.

• Condition 3 was defined as a sham trial with no motor execution (rest–cue–rest) in which the cue period lasted for 10 seconds.

• Condition 4 was a motor execution with no cue period (rest–isometric pinching–rest).

The pinching period was of fixed duration (20 seconds) in all cases. The duration of the rest periods (from 15 to 24 s) was randomized, as was the order of conditions. This allowed for reduced anticipation effects, minimized synchronization of Mayer waves with the motor execution, and stimulated the subject’s concentration. The protocol was split into two sessions of about 20 minutes duration each, with a short break (approx. 10 minutes) in between. Each session started (ended) with a baseline acquisition of 180 (120) seconds.

As no significant influence of the cue periods was observed [34], we processed all rest and pinching trials irrespective of the condition in which they appeared. Taken both sessions together, a total of 30 rest and 30 pinching trials were thus analyzed for the remainder of this paper.

### Data acquisition

#### fNIRS

**Figure 2:**
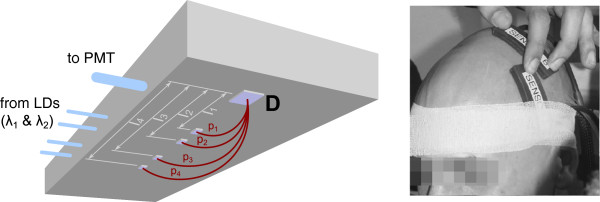
**fNIRS probe.** Left: schematic representation of the fNIRS probe, showing four source sites and one detector site (D). Also shown are optical fibers that guide the light from the laser diodes (LD) and the optical fiber that guides light back to the photomultiplier tubes (PMT). l_1_ to l_4_: source-detector separations corresponding to light paths p_1_ to p_4_. Right: placement of two probes on a subject’s head with adhesive bandages.

Cortical data were obtained with the Oxiplex TS™tissue oximeter (ISS, Inc.). Two probes (Adult Flexible Sensor, ISS, Inc.) were positioned over brain areas involved in the control of right hand movements: (i) left M1, responsible for generating neural commands controlling motor execution; and (ii) left PMv, involved particularly in the control of the force of precision grips [36,37]. The measurement location is referred to with a superscript ^(*L*)^ in the following, with *L*={*M*1,*PMv*}. The positioning was based on the International 10-20 system for EEG electrodes (M1 corresponding to C3, PMv to FC5). To attach the probes, the hair was carefully brushed away to form a parting. The probes were placed such that the optodes were in contact with the scalp and were then fixed to the subject’s head with self-adhesive bandages (Figure 2, right).

The oximeter emitted frequency modulated light at wavelengths *λ*_1_=692 and *λ*_2_=834 nm. A specific wavelength is referred to with a subscript _*λ*_ in the following. For each probe, 4 laser diodes (LD) per wavelength and one photomultiplier tube (PMT) were used. Each probe had four source sites, which received light of both wavelengths from the LDs through optical fibers and one detector site from where light was guided to the PMT through an optical fiber (Figure 2, left). We refer to these four paths with a subscript _*p*_ in the following, with *p*={1,2,3,4}. The collinear source and detector sites were separated by the source-detector distance *l*_*p*_={2,2.5,3.5,4} cm. We used the AC amplitude of the (demodulated) measured light intensity for further processing, as a previous investigation by our group on a phantom showed that it is robust to changes in the ambient light. The 16 raw intensity signals $I_{p,\lambda }^{\left(L\right)}$ (2 locations × 4 paths × 2 wavelengths) were acquired initially at a sampling rate of 50 Hz. The fNIRS data acquisition was controlled by the Oxiplex TS™ software running on a separate laptop PC.

#### Biosignals

**Figure 3:**
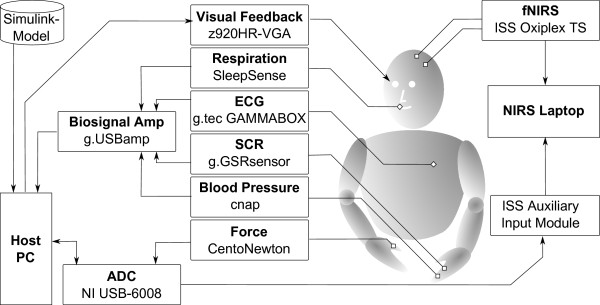
**Schematic representation of the measurement setup.** A Simulink^®^ model was used to run the protocol. The physiological signals (blood pressure, SCR, ECG and respiration flow) were amplified and converted with the signal amplifier, which was connected to the host PC via USB. The force applied by the participant was read by the multifunction I/O-card which also sent trigger-signals for synchronization purposes to the NIRS system. The visual output was provided via VGA to the video-goggles worn by the subject. The two NIRS probes were connected to the NIRS oximeter through optical fibers, and data were sent via USB to the NIRS laptop. Ⓒ2011 IEEE. Reprinted, with permission, from [34].

Our design included the measurement of the blood pressure, respiratory flow, skin conductance response (SCR) and electrocardiogram (ECG). Hence, data from different aspects of the ANS response were obtained, i.e., from the electrodermal (SCR), the cardiovascular (ECG and blood pressure) as well as from the respiratory (respiratory flow) system [38]. All biosignals were fed into the host PC (see Figure 3) via USB using a biosignal amplifier (g.USBamp^®^, g.tec) and were acquired at 600 Hz. 

• ECG: the electrodes (g.GAMMAclip^®^, g.tec) were placed using sticky patches, with ground on left shoulder, reference on left sternum, channel 1 on right sternum, and channel 2 over the left ribs.

• Respiration: the thermistor respiratory flow sensor (SleepSense^®^) was placed in close proximity to the nares, and fixed on the skin with adhesive bandages.

• Blood pressure: the blood-pressure monitor (CNAP monitor 500™, CNSystems) was attached to the left arm by an inflatable cuff, and to the proximal phalanges of the index and middle fingers of the left arm. Subjects were requested to rest the left arm on the chest at the height of the heart.

• SCR: the electrodes (g.GSRsensor™, g.tec) were attached through Velcro^®^ rings to the distal phalanges of the index and middle fingers of the left hand. The skin conductance was acquired by applying a constant micro voltage between the electrodes and measuring the current between the electrodes [39].

#### System integration

The protocol was implemented in Simulink^®^ (The MathWorks^®^, Inc.) running on the host PC. The voltage output of the force sensor was recorded via a USB data acquisition card (NI USB-6008™, National Instruments, Inc.). The same card was used to transmit condition-specific trigger signals which were recorded by the fNIRS system (Auxiliary Input Module, ISS, Inc.). This later allowed for synchronization between the two systems. A schematic representation of the measurement setup is shown in Figure 3.

### Data Pre-processing

The signals from both sessions were imported and further processed off-line with MATLAB^®^ (The MathWorks^®^, Inc.).

#### fNIRS

As the signal components due to changes in cortical activity vary rather slowly (the frequencies present in the canonical hemodynamic response function barely exceed 0.2 Hz [40]) and in order to reduce the computational burden in the subsequent processing steps, the fNIRS signals were downsampled to 5 Hz. To detect motion artifacts (MA), the inverse z-score of the raw intensity signals within a centered sliding window (25% overlap) with a length of 32 data points (6.4 seconds) was calculated, as well as the inverse z-score of the first half of that window. Similar to the method described in [41], a window was considered to contain a MA if the mean ratio (taken across the four light paths of a location-wavelength combination) of these two numbers was greater than a threshold value, which was empirically set to 3 after visual inspection. To prevent false MA detections due to noise, a 5-point median filter was applied prior to the calculation of the inverse z-scores. Trials that were affected by MAs were later excluded from the analysis.

From the raw intensity values, the changes in optical density, *ΔOD*, were calculated at each discrete time point *t*_*k*_,

(1) $$\mathrm{\Delta O}D_{p,\lambda }^{\left(L\right)}(t_{k})=-\ln\left(\frac{\overset{\left(L\right)}{\underset{p,\lambda }{I}}\left(t_{k+1}\right)}{\overset{\left(L\right)}{\underset{p,\lambda }{I}}\left(t_{k}\right)}\right)$$

and converted into changes in oxyhemoglobin (*ΔO*_2_Hb) and deoxyhemoglobin (*Δ*HHb) using the modified Beer-Lambert-Law (MBLL) [42]:

(2) $$\begin{array}{lcr} \;\left[\begin{array}{c} \Delta {O_{2}\text{Hb}}_{p}^{\left(L\right)}\\ \Delta {\text{HHb}}_{p}^{\left(L\right)} \end{array}\right] & = & \frac{1}{l_{p}}{\left[\begin{array}{cc} \varepsilon_{O_{2}\text{Hb},\lambda_{1}} & \;\varepsilon_{\text{HHb},\lambda_{1}}\\ \varepsilon_{O_{2}\text{Hb},\lambda_{2}} & \;\varepsilon_{\text{HHb},\lambda_{2}} \end{array},\;\right]}^{-1}\\ & & \times \left[\begin{array}{cc} \text{DP}F_{\lambda_{1}}^{-1} & \;0\\ 0\; & \text{DP}F_{\lambda_{2}}^{-1} \end{array}\right]\;\left[\begin{array}{c} \mathrm{\Delta O}D_{p,\lambda_{1}}^{\left(L\right)}\\ \mathrm{\Delta O}D_{p,\lambda_{2}}^{\left(L\right)} \end{array}\right] \end{array}$$

with the wavelength-specific differential pathlength-factors $\text{DP}F_{\lambda_{1}}=6.51$ and $\text{DP}F_{\lambda_{2}}=5.86$[43], and the extinction coefficients $\varepsilon O_{2}\text{Hb},\lambda_{1}=0.9556$, $\varepsilon_{O_{2}\text{Hb},\lambda_{2}}=2.3671$, $\varepsilon_{\text{HHb},\lambda_{1}}=4.8538$ and $\varepsilon_{\text{HHb},\lambda_{2}}=1.7891$ (cm mM) ^−1^[44]. The time courses of oxy- and deoxyhemoglobin were obtained by summing up their temporal changes.

After session-wise subtracting the mean and normalizing by their standard deviation [18], the signals were low-pass filtered using a second-order Chebychev type-II filter with an attenuation of 40 dB at 0.5 Hz [34]. This was done to suppress systemic components such as pulsation and respiration from the fNIRS data [45]. To remove signal drift, a high pass filter at 1/54 Hz was employed by truncating the signal’s direct cosine transform and re-converting it back into the time domain. The selected cutoff-frequency corresponded to the longest possible time period in the protocol, and it was assumed that every frequency component below was not elicited by the experimental intervention [34]. The pre-processed fNIRS signals are denoted by ${O_{2}\text{Hb}}_{p}^{\left(L\right)}$ and ${\text{HHb}}_{p}^{\left(L\right)}$.

#### Biosignals

The blood pressure signal was detrended by removing the linear fit from the signal of each session. It was low-pass filtered with a 1st order Butterworth filter with cutoff frequency of 0.1 Hz in order to focus on the low and very low frequency spectra of the signal (called mean blood pressure in the following sections). The selection of the low and very low spectra of the blood pressure was performed after carefully studying which features related to blood pressure could bring the maximum information to the classifier. Although, initially, the diastolic, systolic and raw blood pressure signals were also considered, they were discarded after confirming that the difference of these signals between the execution and rest periods were less significant than the mean blood pressure, denoted here by BP.

The ECG signal was filtered with a 4th order Butterworth bandpass filter with a frequency band of 0.01-40 Hz. The HR was calculated using an adaptive threshold similar to the one described in [46] on the squared derivative signal of the QRS complex. The HR was simultaneously calculated using a similar adaptive threshold on the raw blood pressure in order to increase the HR detection robustness. Only the heart rate was considered as a feature for the classifier, since other time and frequency domain measures of heart rate variability require a time interval of more than two minutes in order to be calculated [47].

The respiration signal, measured by the nasal thermistor flow sensor, was filtered with an 8th order Butterworth bandpass filter with a frequency band of 0.1-2.1 Hz. The breathing rate (BR) was calculated using an adaptive threshold similar to the one applied to the QRS complex, using the signal derivative. The breathing amplitude was initially also considered, but was discarded after confirming that there was no variation in breathing amplitude between the execution and rest periods.

Skin conductance is characterized by a slowly-changing background level (tonic), and a rapid time-varying (phasic) response [39]. The phasic response is what is referred to as conductance response and is usually related to the response to external stimuli. The skin conductance signal was filtered with an 8th order low-pass filter with a cutoff frequency of 30 Hz, and linearly detrended to remove the tonic level over time using the start of each trial as a breakpoint. The SCR data was further normalized.

As performed with the fNIRS signals, all biosignals were then downsampled to 5 Hz, session-wise centered around zero and normalized by their standard deviation.

### Classification and performance

The goal of the classification step was to decode on a single trial basis whether the subjects were executing the motor task or at rest based on the pre-processed signals. Two classification approaches were investigated: (i) decoding based only on fNIRS signals, and (ii) decoding with the combination of fNIRS data and biosignals. Types of classifiers that were shown to be suitable for fNIRS-based BCI applications include SVM, LDA, ANN, and HMMs [18,20,22-24,26,27]. We chose HMMs as the classifier framework because of their ability to capture the dynamic nature of hemodynamic and biosignal time series, and their proven aptitude in classifying fNIRS data [18,20].

HMMs are well known in applications such as speech and gesture recognition (see [48] for a detailed tutorial). Here, only a brief introduction is given. An HMM is a finite state machine composed of a discrete set of *N*_*s*_ unobservable (“hidden”) states. These states form a Markov chain, i.e. the probability of transition to subsequent states only depends on the current one and is described by a transition probability matrix. While the model resides in a certain state, it can emit observable signals (i.e. observations) according to a state-specific observation probability. For a fixed number of states, an HMM is completely described by the starting and transition probabilities and the observation probability distributions at each state. Training of an HMM is carried out by adjusting these model parameters to observations belonging to the *training* dataset, while withheld *test* observations are used to evaluate the trained HMM’s ability to generalize.

HMMs allow for multivariate observations and operate on sequential data. The observations were thus based on several data *segments*, i.e. sections of signals that were recorded during a particular pinching or rest trial. From the fNIRS data, *feature signals*, calculated based on the pre-processed fNIRS data as described below, were extracted as the basis for the fNIRS observations.

#### fNIRS feature extraction

fNIRS features were extracted from the 16 pre-processed fNIRS signals ${O_{2}\text{Hb}}_{p}^{\left(L\right)}$ and ${\text{HHb}}_{p}^{\left(L\right)}$ (4 paths, 2 locations, oxy-/deoxyhemoglobin) to reduce the dimensionality of the observations in the subsequent classification step. A simple but effective approach was employed based on linear combinations [49,50]. The idea was to find a linear combination of all eight signals belonging to the same measurement location *L*, that best fits the time course of the protocol (i.e. is one during pinching periods and zero during rest). The method is adaptive in the sense that it only makes use of training data and hence does not need to be tuned *post-hoc* for individual subjects.

**Figure 4:**
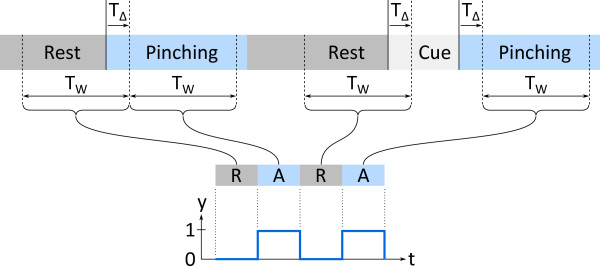
**Illustration of the class-wise segment extraction.** Two rest (R) and two active (A) data segments are shown. *T*_*W*_ : window length, set to 75 data points (15 seconds). *T*_*Δ*_ : temporal shift, set to 25 data points (5 seconds) to compensate for hemodynamic delay. y: binary signal that was used as the target signal for the fNIRS feature extraction.

**Figure 5:**
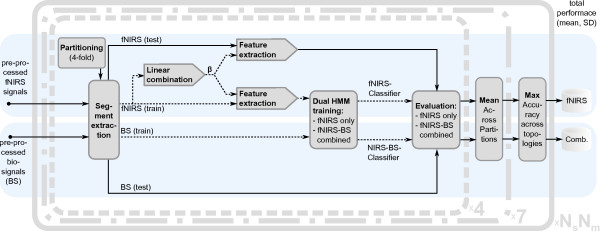
**Overview of the signal flow in the data classification.** Data segments were extracted from pre-processed fNIRS data and biosignals. Training data are indicated with dashed arrows, and test data are displayed with solid arrows. fNIRS training segments were used to extract feature signals based on linear combinations of all signals from one location, and observations were formed based thereon (block “Feature extraction”). Biosignal observations were directly formed from segments of the pre-processed biosignals. The training observations were used to train two dual HMMs either with fNIRS data only or with the combined (fNIRS and biosignals) observation. The trained classifiers were evaluated based on the test observations. The classification results of four different partitions (dashed bounding box) were averaged to yield the mean performance of a complete cross-validation run. This was repeated a total of seven times (dash-dotted bounding box) to yield the mean classification performance for a given model topology. Ten different model topologies were investigated (solid bounding box) and the one with highest performance was selected.

The stimulus onset times for pinching periods and the end points of the preceding rest periods for all training rest-pinching pairs were identified. Based thereon, data segments within a window of a fixed length *N*_*W*_ were extracted (see Figure 4 and block “Segment extraction” in Figure 5). *N*_*W*_ was set to 75 data points (corresponding to *T*_*W*_ =15 seconds, the shortest possible rest interval in the protocol). To account for latencies in the hemodynamic response, the window was shifted forward by *T*_*Δ*_. This shift was set to 5 seconds (corresponding to *N*_*Δ*_ =25 data points), as it has been suggested that a shift of 5 seconds or more is required to account for the hemodynamic delay [25].

Segments were denoted as *active* if the data was recorded while subjects were isometrically pinching, and as *rest* otherwise. Only the *N*_train_ pairs from the training set without MAs were considered for the feature extraction step. These segments were concatenated in random order for each location *L* separately to yield the two matrices ${\hat{M}}^{\left(L\right)}$ (with 8 columns and 2·*N*_*W*_·*N*_train_ data points as rows). A target signal *y* was constructed, containing zeros for data points that belong to rest trials and ones for active trials (Figure 4). Linear weights *β*^(*L*)^ were calculated to separate rest and active segments as

(3) $$\beta^{\left(L\right)}=\underset{\beta }{arg min}\left\{{\left({\hat{M}}^{\left(L\right)}\cdot \beta -y\right)}^{2}\right\}$$

Having hence found a set of linear weights that separate the training trials, the fNIRS feature signals were calculated for both locations as the weighted sum of the original pre-processed fNIRS signals ${O_{2}\text{Hb}}_{p}^{\left(L\right)}$ and ${\text{HHb}}_{p}^{\left(L\right)}$. By that, the dimension of the fNIRS signals was reduced from originally 16 to 2 (one signal per measurement location).

#### Observations

The fNIRS-observations were extracted for each trial based on the two feature signals in the same way as shown in Figure 4, by extracting the portions of the signals in a window of length *N*_*W*_, shifted forward by *N*_*Δ*_ data points. The 2-dimensional (*M*1 and *PMv*) observation for trial *i* of the fNIRS data was denoted by $O_{\text{NIRS}}(i)\in R^{N_{W}\times 2}$. The biosignal-observations were extracted analogously based on all the 4 pre-processed signals (i.e. without further feature extraction steps). The 4-dimensional observation vector (BP, HR, BR and SCR) of the biosignals was denoted by $O_{\text{BS}}(i)\in R^{N_{W}\times 4}$ for the *i*-th trial. Associated rest-active trial pairs that were affected by MAs were excluded from both *O*_NIRS_ and *O*_BS_.

**Figure 6:**
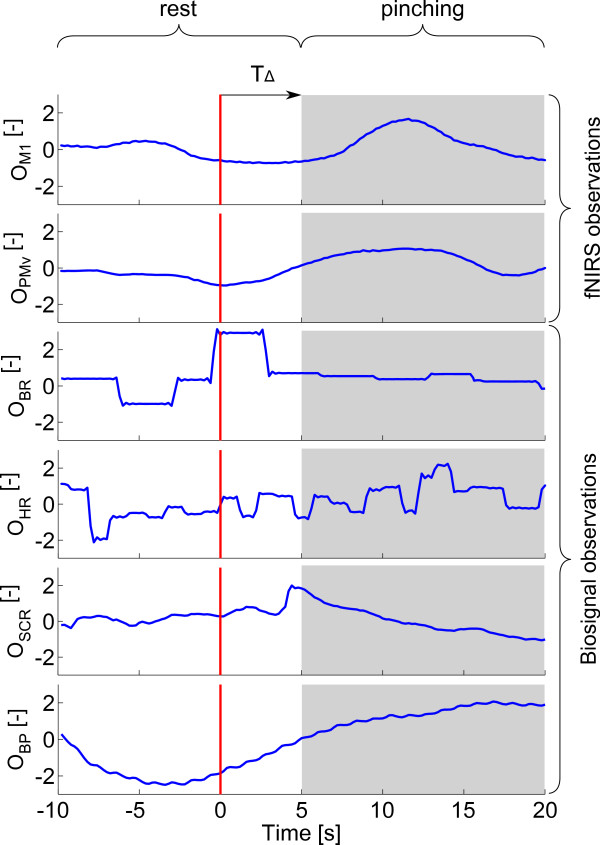
**Representative observation signals.** A rest (white background) and a pinching (gray background) test trial are shown. The red vertical line at *t*=0 represents the stimulus onset when the subject started pinching isometrically, *T*_*Δ*_ is the shift of 5 seconds that was applied for the extraction of observations. *O*_*M*1_, *O*_*PMv*_ : fNIRS observations of data from M1 and PMv, respectively. *O*_*BR*_, *O*_*HR*_ : observations of breathing and heart rate, respectively. *O*_*SCR*_ : observation of skin conductance response. *O*_*BP*_ : observation of blood pressure signal. Note that all observations are unit-less due to the normalization steps in the pre-processing.

Two different approaches to classify rest and pinching periods based on fNIRS and biosignal data were investigated. First, a classifier was trained and evaluated based on NIRS data only, i.e the observation was set to *O*=*O*_NIRS_. The effect of combining fNIRS and biosignals was then analyzed by considering the observation *O*=[*O*_NIRS_*O*_BS_] for training and evaluation. An example of an observation for two associated test trials (one rest and one active) is given in Figure 6 for the fNIRS-biosignal combined decoder.

#### Model training

As the experimental protocol was not *a priori* expected to result in cyclic fNIRS or ANS responses, a left to right Markov model structure was chosen by starting in the leftmost state and allowing transitions from each state to itself and its direct right neighbor. The observation probability distributions were chosen to be mixtures of *N*_*m*_ Gaussians with full covariance matrices and the same number of mixtures for each state. For each subject, ten different model topologies (i.e. all combinations of *N*_*s*_∈{1,2,3,4,5} and *N*_*m*_∈{1,2}) were investigated. A freely distributed HMM toolbox for MATLAB^®^ was used for the analysis described here [51].

All training observations were first grouped using k-means clustering (*k*=*N*_*s*_×*N*_*m*_). The clusters were used to initialize the observation probability distributions, while the state transitions were initialized randomly. The model parameters were optimized using expectation maximization (EM) techniques. One separate HMM was identified per class: *HMM*_*r*_ was trained with the training observations belonging to rest trials, and *HMM*_*a*_ was trained with data from active trials.

#### HMM initialization

Due to the stochastic nature of the initialization procedures, the training of the two models was repeated 10 times. This resulted in 10 candidate models for either class (*HMM*_*a*_ and *HMM*_*r*_). A performance assessment was done for each model combination as follows: for each trial *i* in the training set with observation Ô(i), the logarithm of the likelihoods (*LL*) that the data were generated from either model were calculated, i.e.

(4) $$LL_{a}(i)=\log P\left(Ô(i)\;|\;\text{HM}M_{a}\right)$$

(5) $$LL_{r}(i)=\log P\left(Ô(i)\;|\;\text{HM}M_{r}\right)$$

and used to determine the difference

(6) $$D(i)=LL_{a}(i)-LL_{r}(i)$$

between these two log likelihoods. Each training trial was assigned a class label *c* based on this difference,

(7) $$c(i)=\left\{\begin{array}{cc} \text{active} & \text{if}\;D(i)>0\\ \text{rest} & \text{otherwise} \end{array}\right)$$

which was compared to the (known) actual class of Ô(i). The outcome was stored in the variable *u*, with

(8) $$u(i)=\left\{\begin{array}{ll} \;1 & \;\text{if}\;c(i)\;\text{is correctly classified}\\ -1 & \;\text{otherwise} \end{array}\right)$$

The performance *κ* was then calculated as

(9) $$\begin{array}{lll} \;\kappa & =\frac{\underset{j\in \{n|u(n)=1\}}{\sum }|D(j)|}{\underset{j}{\sum }|D(j)|}\; & \;\\ & =\frac{\text{summed LL-distance of correct trials}}{\text{summed LL-distance total trials}}\; \end{array}$$

Consequentially, *κ* represented a weighted accuracy of the classification of the training data. The procedure was repeated for each of the 100 possible model combinations. The combination $\left\{\text{HM}M_{r}^{*},\text{HM}M_{a}^{*}\right\}$ that maximized *κ* was then selected.

#### Dual HMM classification

The class assignment for a test trial *i* with observation Õ(i) was carried out by simply comparing the log likelihoods that either model produced the data, i.e.

(10) $$LL_{a}^{*}=\log P\left(Õ(i)\;|\;\text{HM}\overset{*}{\underset{a}{M}}\right)$$

(11) $$LL_{r}^{*}=\log P\left(Õ(i)\;|\;\text{HM}\overset{*}{\underset{r}{M}}\right)$$

If $LL_{a}^{*}$ was higher than $LL_{r}^{*}$, we classified the trial as *active*, otherwise as *rest*:

(12) $$\text{class}=\left\{\begin{array}{ll} \text{active} & \text{if}\;LL_{a}^{*}>LL_{r}^{*}\\ \text{rest} & \text{otherwise} \end{array}\right)$$

### Assessment of classification performance

A 4-fold cross-validation was used to randomly split all pairs of associated rest-pinching trials into 4 partitions that comprised the training and the test set (75% and 25% of the data, respectively). The pairwise partitioning resulted in the same number of rest and pinching trials in each set. In the binary classification presented here, four outcomes were possible: a pinching trial was correctly classified as pinching (true positive, *tp*), a rest trial correctly classified as rest (true negatives, *tn*), a pinching trial wrongly classified as rest (false negatives, *fn*) and a rest trial wrongly classified as pinching (false positives, *fp*). The classification performance was assessed for a given partition by summing up the outcomes for each trial in the test set (i.e. TP=∑tp, TN=∑tn, FN=∑fn, FP=∑fp), and computing the classifier accuracy (*Acc*), sensitivity (*Sens*) and specificity (*Spec*) as follows:

(13) $$\text{Acc}=\frac{\text{TP}+\text{TN}}{\text{TP}+\text{TN}+\text{FP}+\text{FN}}$$

(14) $$\text{Sens}=\frac{\text{TP}}{\text{TP}+\text{FN}}$$

(15) $$\text{Spec}=\frac{\text{TN}}{\text{TN}+\text{FP}}$$

The above assessment was carried out for every partition, i.e. 4 times. The mean accuracy, sensitivity and specificity of these 4 partitions were calculated as the performance of one complete cross-validation run. Due to the stochastic nature of the HMM training and in order to reduce the effect of variance due to the random selection of test and training samples during the partitioning, this procedure was repeated 7 times. The mean and the standard deviation of accuracy, sensitivity and specificity of these 7 complete cross-validation runs were computed. From all possible model topologies, i.e. combinations of *N*_*s*_ and *N*_*m*_, the one with the highest mean accuracy was then selected for each subject individually (Figure 5). This was done separately for both types of classifiers, i.e. for the fNIRS-only decoder as well as the fNIRS-biosignal combination.

### Data analysis

Whether the obtained classification accuracy was higher than chance was tested at the 5% significance level. To this end, the mean accuracy across the seven iterations together with the number of included trials was used to compute the mean number of successfully classified trials. Based on this, the 95% confidence interval (CI) of the accuracy was calculated from a binomial reference model (MATLAB^®^: binofit). Classification results were regarded as significantly different from chance if the CI did not contain the chance level (50%).

The performance differences of the two classifiers were tested for each subject at the 5%-significance level with paired, two-tailed t-tests using the results of each of the 7 complete cross-validation runs. Similarly, the performance difference was tested at the group level with paired, two-tailed t-tests using the average across the 7 cross-validation runs.

All mean accuracies were compared to the reference value of 70%, which has been identified as a lower bound for proper device control and user-friendly operation [52,53].

## Results

All subjects were able to understand the task and completed the experimental protocol, and no subject reported any discomfort during the experiment. Due to technical problems in the recording of the biosignals, only fNIRS data were obtained in the second session for subject 6 and in both sessions for subject 1.

**Figure 7:**
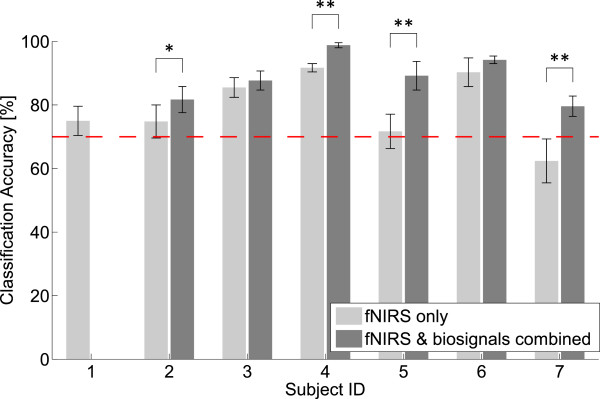
**Classification accuracies of the two different classifiers.** Shown are the classification accuracies (mean ± SD) for fNIRS only (light gray) and the fNIRS-biosignal combination (dark gray) for subjects 1 to 7. The number of states and mixtures were individually chosen to maximize the accuracy. The dashed line indicates 70% accuracy, which was identified as the lower bound for useful BCI operation. ^∗^ : p < 0.05, ^∗∗^ : p < 0.01 (paired t-test across 7 complete cross-validation runs).

We selected the combination of the number of states *N*_*s*_ and the number of mixtures *N*_*m*_ that yielded the maximum accuracy and report the classification performance based on these models. The classification accuracies for all subjects are shown in Figure 7. Details are shown in Table 1, where the mean accuracies, sensitivities and specificities are given for each subject and at the group level for both types of observations. The change in performance when including biosignals is further given in percent increase relative to the fNIRS only decoder, as well as the p-values of the statistical tests on the difference between the two approaches. Note that for subject 6, results for the fNIRS only case are solely based on session 1. In the following, we report results for each classifier separately.

**Table 1:** Detailed comparison of the two classifiers

	**Subject**	**1**	**2**	**3**	**4**	**5**	**6**^**2**^	**7**	**Average**
NIRS only	Acc. ± SD [%]	(75.0 ± 4.6 ^*†*^)	74.8 ± 5.2 ^*†*^	85.5 ± 3.1 ^*†*^	91.7 ± 1.3 ^*†*^	71.7 ± 5.4 ^*†*^	90.3 ± 4.5 ^*†*^	62.4 ± 6.9	79.4 ± 11.7
							(89.7 ± 3.5 ^*†*^)		(78.7 ± 10.6)
	Sens. ± SD [%]	(73.3 ± 5.4)	72.3 ± 5.7	85.7 ± 2.9	88.7 ± 1.5	72.8 ± 6.7	87.2 ± 7.6	62.8 ± 11.6	78.3 ± 10.5
							(89.9 ± 6.3)		(77.9 ± 10.2)
	Spec. ± SD [%]	(76.7 ± 4.9)	77.2 ± 7.6	85.2 ± 5.6	94.8 ± 2.7	70.6 ± 5.1	93.5 ± 4.2	61.9 ± 6.8	80.5 ± 13.0
							(89.6 ± 5.8)		(79.4 ± 11.3)
	*N*_*s*_	(1)	4	1	1	1	3 (3)	2	
	*N*_*m*_	(1)	1	1	2	1	1 (2)	1	
Combined	Acc. ± SD [%]		81.7 ± 4.1 ^*†*^	87.7 ± 3.0 ^*†*^	98.8 ± 0.8 ^*†*^	89.2 ± 4.5 ^*†*^	94.2 ± 1.2 ^*†*^	79.6 ± 3.2 ^*†*^	88.5 ± 7.3
	Sens. ± SD [%]		82.1 ± 4.8	85.6 ± 3.8	99.6 ± 1.2	84.7 ± 8.3	93.8 ± 0.0	77.3 ± 5.1	87.2 ± 8.1
	Spec. ± SD [%]		81.3 ± 7.1	89.8 ± 4.3	98.0 ± 1.9	93.7 ± 5.4	94.6 ± 2.4	81.9 ± 3.3	89.9 ± 6.9
	*N*_*s*_		5	3	5	3	1	3	
	*N*_*m*_		1	2	2	2	1	2	
	Acc. Gain [%]		9.2	2.6	7.7	24.4	4.3	27.6	12.6 ± 10.7
	Sens. Gain [%]		13.6	-0.1	12.3	16.3	7.6	23.1	12.1 ± 7.9
	Spec. Gain [%]		5.3	5.4	3.4	32.7	1.2	32.3	13.4 ± 14.9
	p-value^1^ Acc.		0.016 ^∗^	0.129	< 0.001 ^∗∗^	0.001 ^∗∗^	0.051	0.001 ^∗∗^	0.02 ^∗^
	p-value^1^ Sens.		0.003 ^∗∗^	0.988	< 0.001 ^∗∗^	0.028 ^∗^	0.062	0.034 ^∗^	0.008 ^∗∗^
	p-value^1^ Spec.		0.349	0.089	0.023 ^∗^	< 0.001 ^∗∗^	0.535	< 0.001 ^∗∗^	0.062
	*N*_*Trials*_	(60)	46	48	60	46	30 (60)	58	

Results of the binary classification of solely fNIRS data (upper part) and the combination of fNIRS data and biosignals (lower part). Reported are mean accuracy (Acc.), sensitivity (Sens.) and specificity (Spec.) with SD across 7 complete cross-validation runs for each subject. Further, average values across all subjects are given. For subject 6, session 2 and both sessions of subject 7, biosignals were not available. Numbers in parentheses are shown for completeness and were not used in the comparison between the two types of decoders. *N*_*s*_ : Number of states in the HMM. *N*_*m*_ : Number of Gaussians used to model the HMM’s emission probabilities. Gain: Relative change with respect to the fNIRS only case. *N*_*Trials*_ : total number of trials used for analysis (rest and active). ^1^: Paired t-tests on differences between the fNIRS only and the combined decoders on individual level (difference on group level for averages). ^2^: In brackets are the results when considering both sessions for the fNIRS only decoder. ${}^{*}$ : p < 0.05. ${}^{**}$ : p < 0.01. ${}^{†}$ : Accuracy significantly above chance.

### fNIRS only

Data from 6 of the 7 subjects were classified significantly above chance level. The CI for the accuracy for subject 7 was [48.7,74.8] and hence we could not reject the hypothesis that this result was obtained by chance at the 5% significance level. The mean classification accuracy across all subjects was 79.4 ± 11.7%, the mean sensitivity was 78.3 ± 10.5% and the mean specificity was 80.5 ± 13.0%. All subjects but one (subject 7) showed an accuracy above 70% for the fNIRS only case. The best performing model topology (i.e. number of states *N*_*s*_ and number of mixtures *N*_*m*_) is reported for each subject in Table 1.

### fNIRS and biosignals combined

The inclusion of the biosignals increased the classification accuracy in all subjects, reaching significance for four participants (Figure 7). Data from all subjects were classified well above chance level (Table 1) and the accuracies were all above 70% (Figure 7). At the group level, the inclusion of the biosignals significantly increased the accuracy to 88.5%, 12.6% more than with the fNRIS only classifier (p=0.02, Table 1), the sensitivity significantly increased by 12.1% to 87.2% (p=0.008, Table 1) and the specificity increased by 13.4% to 89.9% (p=0.062, Table 1).

## Discussion

This paper investigated the feasibility of detecting and classifying movement execution in seven healthy subjects performing an isometric pinching task using hemodynamic data and biosignals. Brain hemodynamics measured with functional near-infrared spectroscopy in primary motor cortex and ventral premotor cortex, as well as the ANS response (heart rate, mean blood pressure, breathing rate and skin conductance response) acquired simultaneously were used to train classifiers based on hidden Markov models. Two different decoding approaches to distinguish between rest and active states of the subject were investigated. First, a classifier based exclusively on fNIRS data was trained and evaluated. Secondly, a classifier using a combination of the fNIRS data and biosignals was investigated and compared to the fNIRS only classifier. A comprehensive description of the methodology used to process and analyze recorded data was presented.

In the proposed classifier, no direct motor output parameters (such as force, displacement or EMG activity) were used, resulting in a classification based purely on the cortical processing of motor execution and the related response of the ANS. Although also motor imagery has been used to elicit classifiable signal changes in fNIRS-based BCI studies [18,19,25], an overt motor task was chosen for mainly two reasons. First, it was found that motor execution and imagery activate similar neural structures [54-56], however the data shown in [25] indicate that fNIRS signal changes are generally weaker during imagery. Second, motor execution allows for easier control and assessment of the subject’s behavior and performance. It is known that the ability to perform motor imagery can vary among subjects [54]. Furthermore, different imagery strategies can lead to inconsistency in the obtained data [18].

### Classification of sole fNIRS data

The results for the fNIRS-only decoder indicated that the measured hemodynamic data contain information about whether or not the subjects were in pinching or rest states and can be used for classification purposes. This was expected as it has been previously shown that fNIRS data from motor areas obtained during overt motor execution yield well classifiable signals: in [18], left and right finger tapping was classified with an accuracy of 93.4%. In this study, by comparison, fNIRS signals were classified with a lower accuracy (79.4%). While a 3-wavelength, 20 light path NIRS system was used in [18], the Oxiplex TS™oximeter used here featured a total of 8 light paths and 2 wavelengths. As the positioning with the 10-20 system is only approximative, increasing the number of light paths results in a higher chance to actually reach the activated cortical region, which positively affects the classification accuracy. Furthermore, our approach allows for the classification of rest versus active states, as opposed to left and right hand motor tasks. Ultimately, the explicit detection of resting phases might ease BCI operation, as it does not require subjects to constantly be in an activated state, but also allows for recreation between active phases. Compared with fNIRS BCI studies that employed more mental tasks such as music imagery, mental arithmetic, emotional induction tasks or subjective preference tasks, our results are also in good agreement [20,22-24].

### Classification of combined fNIRS data and biosignals

Our results show that in healthy subjects, hemodynamic data in combination with biosignals as altered by motor execution can be automatically decoded with an average accuracy of 88.5%. This is well above the threshold of 70%, which was identified as the lower bound for proper device control and user-friendly BCI operation [52,53].

The inclusion of biosignals significantly increased the classification performance. The highest gains in accuracy were observed for the subjects that showed the worst performance for the NIRS only decoder (e.g. approximately 25% gain in accuracy for subjects 5 and 7). The positive effect of including biosignals in the classification has been reported for fNIRS measurements in the frontal cortex in [22]. Here, we report for the first time classification performance of a hybrid fNIRS-biosignals classifier that is based on fNIRS data from motor regions. We observed similar gains in performance as were identified in [22].

fNIRS is not exclusively sensitive to changes in cortical activity, but the signals also contain systemic components. Moreover, the isometric finger pinching task influences the ANS. Indeed, when focusing exclusively on the ANS portion of the dataset used in this work, a similar classification analysis yielded encouraging performance, which indicated that the biosignals contain considerable information about whether subjects are at rest or pinching [57]. Two speculations to explain the observed beneficial effect of including biosignals are given here. 

• First, the addition of the ANS response to the classifier, as provided through the biosignals, enhanced the information content about the subject’s state. The classifier had access to not only activity changes in motor areas as measured with fNIRS, but also the autonomic consequences of the experimental task. This multimodal information allowed for a more accurate classification compared to the fNIRS only case.

• Second, the HMM was able to compensate for systemic signal components in the fNIRS data by making use of the biosignals. As full covariance matrices were used to model the emission probabilities, possible correlations between signals were accounted for. This essentially resulted in an improved estimation of the subject’s cortical state and hence in increased classification accuracy.

These two possible explanations are likely to be complementary and might both have led to the observed beneficial effect of including biosignals. However, the information content in the fNIRS data that can be explained by biosignals and vice versa was not systematically investigated in this paper. Further research is needed to study the amount of information in the fNIRS data that is due to physiological effects and positively affects the classification accuracy.

### Study limitations

The present study suffers from some limitations. The inherent noise in the acquired fNIRS signals required considerable steps of pre-processing to filter the data, which could strongly influence classification accuracy. One possible solution to better filter fNIRS signals would be to use the measured biosignals, e.g. heart rate, blood pressure and respiration flow to remove part of the physiological noise contained in hemodynamic signals.

The usage of biosignals increased the requirements on the equipment and the setup time that was needed to attach the sensors significantly. For a therapeutic application, this might be a crucial factor. The usage of biosignals (especially blood pressure) also hampers the option of a wirelessly operated system, which would be feasible with wireless fNIRS probes [15] only. Furthermore, as the number of acquired signals increases, the chance of losing one due to technical reasons increases as well. This might degrade the classification results, but also makes it more difficult to compare results between subjects.

As it is common for most of the fNIRS BCI studies we are aware of, the placement of the fNIRS probes was based on the international 10-20 system. Although this gave an approximative indication about where to place them, some inter-subject variability in the positioning is expected. The inclusion of prior knowledge about the exact location of motor areas could increase the fNIRS signal quality, e.g. through a preceding fMRI screening or a functional localization based on transcranial magnetic stimulation (TMS). We further experienced high dependency between signal quality and the careful placement of the probes. For a day-to-day rehabilitation to be most efficient, the placement needs to be facilitated and made more robust. Further work also needs to be done to identify the optimal probe positioning in stroke patients with lesions in the respective brain areas.

Furthermore, data were acquired in a laboratory setting, i.e. in a dimmed room with minimized external disturbances. Motion artifacts were reduced by the isometric task and by asking the participants to lie on a stretcher. The isometric pinching task was simple and standardized. For a BCI to be effective, however, it is required to process a variety of different motor tasks and be robust against environmental influences as well as artifacts. An increase in the robustness of a HMM-based classifier against auditory startle stimuli has been proposed in [22], where biosignals and fNIRS data were successfully classified in the presence of environmental distraction stimuli by using a technique termed “environmental sniffing”.

As this study was carried out with healthy subjects performing overt motor execution, it remains to be shown to what extent these results can be transferred to a different population, such as patients with neurological injury, which might exhibit altered fNIRS and ANS responses, or may not be able to activate motor-related networks. Additionally, the limited number of subjects does not allow for a direct population-wise generalization of the results, which was not the objective of this feasibility study. Nevertheless, these results can be used as first evidence that the proposed approach is worth pursuing: in six out of seven subjects, HMMs classified fNIRS data from motor areas with an accuracy significantly above chance, and in six of the seven subjects, the mean accuracy was above 70%. Furthermore, in all the participants from whom biosignals were available, their inclusion increased the classification accuracy, with all subjects showing accuracies well above 70%.

### Future work

#### Towards an on-line decoder with user feedback

Useful BCIs must meet some requirements: ultimately, the decoding has to be done on-line and in real-time. We are aware of only one study employing fNIRS for on-line BCI purposes [19]. They used motor imagery in a protocol which was designed to obtain the user’s hemodynamic response to two stimuli. The binary on-line decoding was achieved by comparing the two responses, thereby reaching an information transfer rate of one bit per minute. In this work, we abstained from explicitly using processing steps that are in principle not feasible on-line. Thus, the proposed methods have the potential to be implemented in an on-line decoder, allowing for different forms of on-line feedback to the subject. User feedback has been shown to increase the classification performance: there exists evidence that the signal to noise ratio (SNR) can be increased after training with on-line visual feedback [58]. In a EEG-based BCI study, it was further shown that haptic feedback facilitates the decoding of movement intention in healthy subjects [59].

#### Minimizing classification delay

The signal segments forming the different observations for each trial were 15 seconds in duration. Furthermore, the segments were shifted ahead by 5 seconds. Considering the stimulus onset times in the protocol, this would lead to a maximum delay of 20 seconds. In an fNIRS based motor imagery study where window lengths were selected such as to maximize classification accuracy, the average analysis time intervals were approximately 7 seconds long [25]. Although the peak of the hemodynamic response is known to be delayed by 5 to 8 seconds after the stimulus onset [20], it was shown that this delay can be reduced by 50% with a careful selection of features [60]. Alternative approaches to decrease detection delays include the reliable detection of the fast optical signal [61] or to focus on the initial dip in the hemodynamic response [62].

#### Decreasing training time

A practical BCI should have a short tuning time (training data acquisition and classifier training). The individual tuning of hyper-parameters (such as window lengths, time shifts, type of features, employed classifier and model topology) based on the whole available data set at the subject-level is time consuming. We therefore restricted ourselves to select the most informative fNIRS features solely based on training data and fixed the timing for the observation extraction at the group level, instead of iterating over these parameters. Hence, we may need a rather short time period from the first data acquisition to operational BCI control in a naïve subject. This is a major advantage compared to EEG-based BCIs, where users often need long training periods to be finally able to produce classifiable signals.

#### Potential in stroke rehabilitation

Based on the hypothesis that neuroplastic changes can be induced by providing sensory stimulation through robotic assistance simultaneously with the activation of the motor system [3,59,63], an interface based on the proposed algorithms developed in this paper could have the potential to be employed in the context of motor rehabilitation after neurologic injury. To this end, however, we ultimately need to decode mental tasks that do not involve overt movement but at the same time activate motor regions. One option is to perform motor imagery to trigger assistance, since it elicits similar activation patterns in the human brain as overt execution [25,54-56]. Indeed, recent findings provide evidence that the combination of cognitive aspects of motor control–such as motor imagery–and sensorimotor training can have beneficial effects on the reorganization of the brain after traumatic brain injury [64].

## Conclusion

This study showed for the first time classification results with hemodynamic data obtained from motor regions and simultaneously acquired biosignals. We confirm that HMMs are capable of classifying fNIRS data as well as their combination with biosignals, thereby reaching respectable performance. The combination of the two modalities increased the classification accuracy significantly by 12.6% compared to fNIRS classification only. These results are a promising step towards the development of a brain-computer interface for rehabilitation, with the potential to provide new solutions for the treatment of severely impaired patients.

## Competing interests

The authors declare that this work was done in absence of competing interests.

## Authors’ contributions

RG, MW and RR contributed to the experimental design and project supervision. RR had the idea of increasing fNIRS prediction accuracy by including biosignals. MCF participated in the study design and data acquisition. JE further contributed to the data processing and analysis. RZ, LMC and OL contributed to all these aspects and prepared the manuscript. All authors read and approved the final manuscript.
